# Evaluation of the effects of the low-level laser therapy on swelling, pain, and trismus after removal of impacted lower third molar

**DOI:** 10.1186/s13005-016-0121-1

**Published:** 2016-07-26

**Authors:** Hilal Alan, Ümit Yolcu, Mahmut Koparal, Cem Özgür, Seyit Ahmet Öztürk, Sıddık Malkoç

**Affiliations:** 1 Department of Oral and Maxillofacial Surgery, Inonu University, Faculty of Dentistry, Malatya, 44280 Turkey; 2 Department of Oral and Maxillofacial Surgery, Faculty of Dentistry, Adıyaman University, Adıyaman, Turkey; 3 Department of Ortodontics, Faculty of Dentistry, Inonu University, Malatya, Turkey

**Keywords:** Laser, 3rd molar, 3dMD, Pain, Swelling, Trismus

## Abstract

**Background:**

In current study we aimed to examine the effect of a low-level laser therapy on the pain, mouth opening and swelling of patients whose impacted 3rd molar tooth was extracted in addition measurement volumetrically to the edema with 3dMD face system.

**Methods:**

It was surveyed 15 patients who had bilateral symmetric lower 3rd molars. Surgical sides of patients were randomly separated into two groups: the study group and the control group. It was applied extra oral low-level laser therapy (LLLT, 0.3 W, 40 s, 4 J/cm^2^) to the study group (*n* = 15) after the surgical operation and on the 2nd day. Only routine postoperative recommendation (ice application) was made in the control (*n* = 15) group. The maximum mouth opening, pain level and facial swelling evaluated. 3dMD Face® (3dMD, Atlanta, GA) Photogrammetric System was used to evaluate volumetric changes of the swelling.

**Results:**

There was no statistically significant difference in the edema and interincisal opening between the groups and the pain level in the laser group was significantly lower than in the control group on the 7^th^ postoperative day.

**Conclusions:**

Although there were decreasing trismus, swelling, and pain level, with this LLLT, there was significant difference only in the 7th day pain level in the laser group compared with the control group.

## Background

The most frequently performed surgical procedure in maxillofacial surgery is impacted 3rd molar tooth extraction. It is a minor surgical procedure practiced mostly under local anaesthesia; difficulty varies according to the location of the tooth [1]. The anatomical location wound surface of the surgical area is in the mouth, and the area is constantly irritated due to the movement of the mouth, which may lead to postoperative complaints among patients rather than the surgery itself [2]. Patients’ main clinical complaints are postoperative pain, swelling, and limited opening of the mouth. To prevent these complaints, researchers have suggested many methods such as administering preoperative systemic and topical anti-inflammatory drugs and applying laser therapy [3–5].

Lasers are effectively used in dentistry as well as widely used in many areas of medicine. In dentistry, lasers are generally used in such practices as the treatment of aphtha, fracture healing, gingivoplasty, gingivectomy, frenectomy, biostimulation of soft and hard tissue wound, echodentography and dental imaging. [6–11].

Researchers have determined that laser therapy has analgesic, anti-inflammatory, and biostimulant effects, increases tissue nutrition and connective tissue elasticity, reduces edema, increases lymphatic drainage, and increases regeneration in the synovial membrane [12].

The effect of laser therapy depends on the wavelength and the dosage of the laser beam [13–19]. Low-energy lasers generally have less than 90 mW power. These lasers should be distinguished from the high-energy (10–100+ W) lasers used in surgery, dermatology, and ophthalmology. Low-dosage lasers emit the lowest level of energy and are a type of intensive, focal light therapy [20]. These lasers are also used for “biostimulation” at low dosages in tissue. Low-dosage lasers accelerate wound healing especially in diabetic patients by stimulating fibroblast proliferation with wavelengths ranging between 300 and 400 mw/cm^2^ [21, 22].

With the developing technology, the edema that occurs in patients can be numerically determined through computer systems. In this study, the three-dimensional (3D) photographic image method 3dMD Face® (3dMD, Atlanta, GA) was used to help quantify postoperative volumetric changes after removal of wisdom third molar. Several studies have shown the accuracy and reproducibility of the 3D imaging technique to measure facial appearances [23]. In 1944, Thalmaan was the first researcher to use the stereophotogrammetry technique in clinical studies [24]. Photogrammetry finds a point on a surface in space. Stereophotogrammetry is a more complex technique that provides the 3D coordinates of an object in space. In this method, depth information of the points in images from different cameras is obtained based on the distance from specific measurement areas. The location of all points on an object on the x, y, and z axes in space is given by the computer program. The object is thus called the point cloud. Then the point clouds are combined, and a wire cage-like view called a wireframe is obtained. The surface texture is obtained by covering this cage with a color photograph [25]. Data obtained using these views provide the opportunity to more clearly measure the edema and postoperative changes that occur.

Although several studies have evaluated the efficiency of LLLT in preventing swelling, trismus and pain after the removal of impacted 3rd molars, there are still conflicting results of effect of LLLT on the edema, swelling and pain. However, in the literature there is no study that measured to edema with 3dMD face system. So, in this study we aimed to examine the effect of a LLLT on the pain, mouth opening and swelling of patients whose impacted 3rd molar tooth was extracted in addition measurement volumetrically to the edema with 3dMD face system.

## Methods

Fifteen patients with asymptomatic bilateral wisdom mandibular 3rd molar participated in the study. Patients who had bilateral impacted, III B surgical difficulty grade and required the removal of lower 3rd molars in symmetrical position were included the study. The exclusion criteria included contraindications of laser therapy, systemic illness, current smoking habit, local infection, acute pericoronitis, pregnancy, or breastfeeding. All subjects were informed of the risks of oral surgery and empirical treatment, and they signed a consent form approved by the institution.

Surgery was performed under local anesthesia with 2 ml of 4 % articaine with epinephrine 1:100,000 (Ultracain® D-S Forte, Sanofi Aventis, Istanbul, Turkey) in two sessions separated by at least a month. A single surgeon performed all surgical procedures in order to avoid differences among different surgeons’ skills, which might have influenced the results. All patients required a similar surgical technique for both procedures, because the two 3rd molars were symmetric and had a similar degree of difficulty. A random side impacted tooth of the patients was extracted at the first appointment, and an extraoral laser was applied on the area of masseter muscle immediately after the surgical procedure and at the appointment 2 days after the surgical procedure. At the follow-up appointment 1 month later, it was determined that the patients had achieved their normal mouth opening, and there was complete healing in the area of the surgical procedure. The other side impacted 3rd molar tooth was then extracted. Ice was applied for the first 48 h.

After each surgical procedure, 500 mg paracetamol (Parol, Atabay, Istanbul, Turkey) and benzydamine HCL + chlorhexidine gluconate gargle antiseptic solution (Farhex, Santa Farma, Istanbul, Turkey) were administered two times per day for 7 days. All patients were advised not to use ice after the surgical procedure on the laser-applied side, to control the impact of the laser on facial edema.

In this study, a gallium–aluminum–arsenide (GaAlAs) diode laser device (CHEESE Dental Laser System, Wuhan GigaaOptronics Technology Company, China) with a continuous wavelength of 810 nm was used, and the laser therapy was applied by using a 1 × 3-cm hand piece with non-contact mode. Laser energy was applied to treatment group at 300 mW (0.3 W) for a total of 40 s. Patients in the low-level laser therapy (LLLT) group (*n* = 15) received 12 J (4 J/cm^2^) low-level laser irradiation at the insertion point of the masseter muscle immediately after the surgery and the 2nd day after the surgery.

The variables evaluated were gender, age, facial swelling, length of the surgical procedure, level of pain degree, and the maximum mouth opening. The length of the surgical procedure was defined as the time between the incision and the last suture.

The pain levels were recorded on the visual analogue scale (VAS) of 10 cm; the scores ranged from 0 (no pain) to 10 (the worst pain possible). The pain was recorded after surgery, 2nd day, and 7th day always at the same time. Mouth opening and facial swelling were recorded three times: before surgery, after 2 days, and after 7 days.

The maximum mouth opening (MMO) was determined by evaluating the interincisal distance with a compass, and facial edema was determined with 3D images of the patients.

**Fig. 1:**
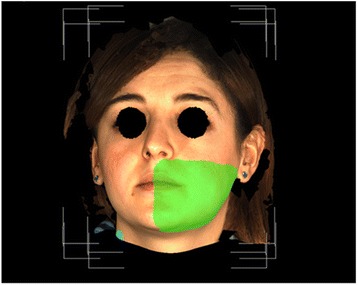
Selected area for measuring of swelling

**Fig. 2:**
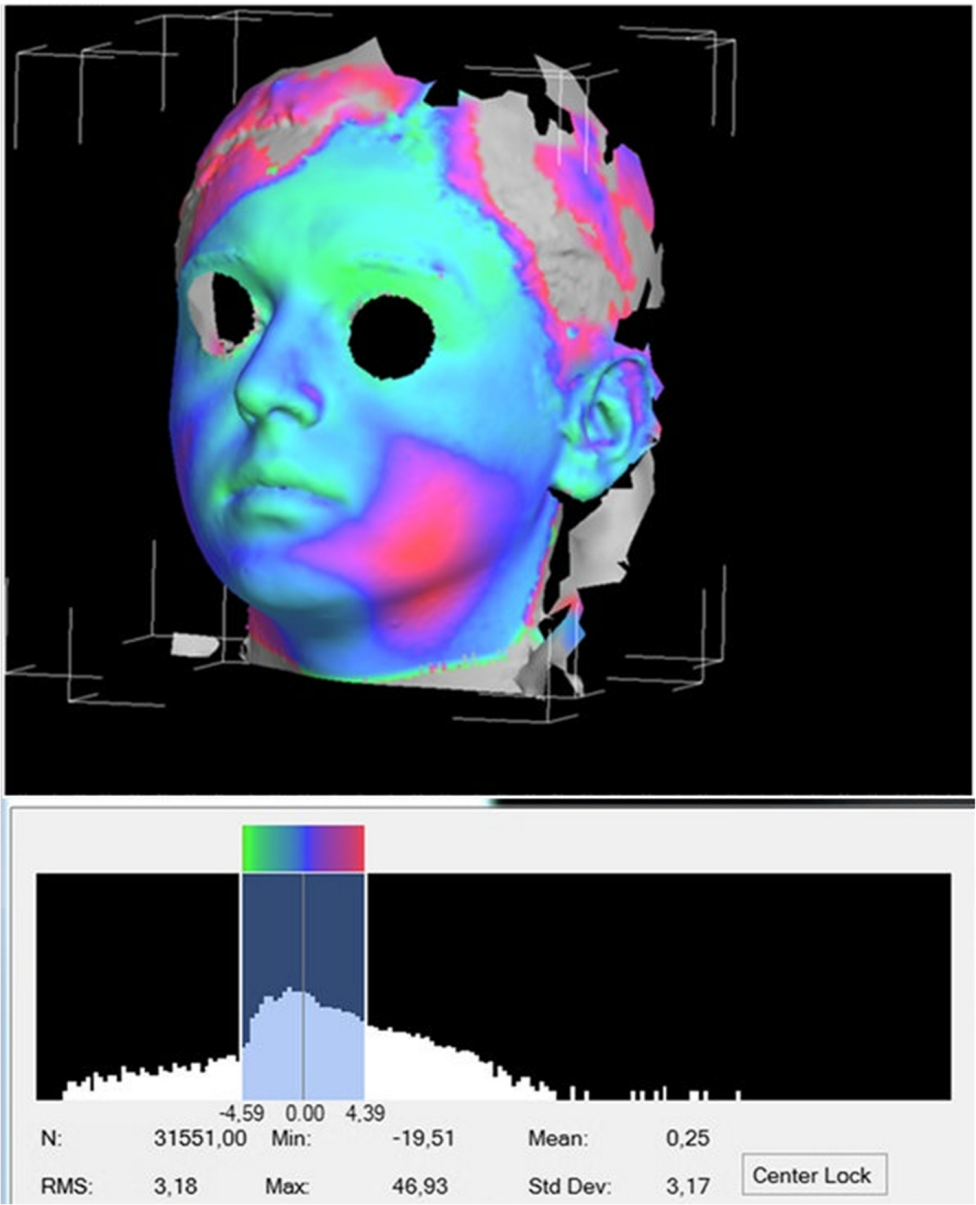
Histogram image of swelling

The 3dMD Vultus program (3dMD, Atlanta, GA) was used to analyze the images. In this program, two different images can be aligned on the chosen surfaces. Linear and volumetric measurement can be made between the aligned images. The analysis began by transferring the records of the patients taken before the surgical procedure (T0), 2 days (T1) after the surgical procedure, and 7 days (T2) after the surgical procedure as a.tsb document to the Vultus program. Two images were aligned on the forehead and nasofrontal area in order to examine them after the images were adjusted. A quadrilateral area with the subnasale, tragion, gonion, and menton points as the corners was selected after the images were aligned (Fig. 1), and the volumetric difference between the two surfaces was measured by calculating the volumetric difference (Fig. 2).

IBM SPSS statistics 22.0 program was used in the statistical assessment. The data were summarized as the smallest and the biggest with the median. The compliance of the data with the normal distribution was assessed using the Shapiro-Wilcoxon test. The Mann–Whitney *U* test was used to compare the two groups. *p* < 0.05 was considered significant.

## Results

The patients’ average age was 22.58 (17–29). Duration of surgery was similar between the laser and control groups (*p* > 0.05; see Table 1). The patients experienced no side effects of the applied treatment.

**Table 1:** Duration of surgery

	Group 1	Group 2	
	Mean ± SD	Mean ± SD	*p*
Duration of surgery	12,9 ± 3,8	13,0 ± 4,0	0,959

There was a small decrease in pain intensity in the side on which LLLT was applied on the 2^nd^ and 7^th^ day after surgery; however, there was a statistically significant difference only on the 7^th^ day (*p* < 0.05; Table 2).

**Table 2:** Evaluation of VAS, swelling, and interincisal opening

		Laser group	Control group	
		Mean ± SD	Mean ± SD	*p*
Edema	2^nd^day	24,1 ± 16,2	21,1 ± 12,9	0,618
	7^th^day	3,6 ± 3,9	5,6 ± 7,8	0,485
	p	**0,002**	**0,002**	
VAS	2^nd^day	4,8 ± 1,9	5,4 ± 1,7	0,440
	7^th^day	**1,3 ± 1,2**	2,7 ± 1,2	**0,017**
	p	**<0,001**	**0,001**	
Mouth opening	0 day	45,5 ± 4,1	43,2 ± 5,5	0,250
	2^nd^day	26,3 ± 4,7	25,0 ± 3,3	0,430
	7^th^day	39,0 ± 5,2	36,0 ± 4,1	0,129
	p	*p* <** 0,001**	*p* <0,001	

Bold characters in each column represent the differences between the measurement periods

For facial swelling, although the laser group had less swelling than the control group on the 2nd day, there was no significant difference between the two groups after the 2nd and 7th postoperative days (*p* < 0.05; Table 2).

The LLLT group presented a greater degree of oral opening than the control group on the 2nd and the 7th day after surgery, but there was no statistically significant difference (*p* > 0.05; Table 2).

## Discussion

The present study examined the effect of LLLT on swelling, pain, and trismus after lower 3rd molar extraction. In this study, we found that although the laser group had less swelling and a greater degree of oral opening there was no statistically significant difference (*p* > 0.05). There was a statistically significant difference only on the 7th day in the decrease in pain intensity (*p* < 0.05).

It was determined that laser application has analgesic, anti-inflammatory, and biostimulant effects, increases tissue nutrition and collagen tissue elasticity, reduces edema, increases lymphatic drainage, and increases regeneration in the synovial membrane [12, 26, 27]. The biostimulation effect of LLLT is controversial. The absence of constant parameters of physical and biological variables of the lasers applied in a previous study, for instance, the type of laser, frequency of pulse, output of power, time of application, wavelength, and distance of the source from the tissue, cause difficulties in calibrating the results [28].

Although several studies have evaluated the efficiency of LLLT in preventing swelling and trismus after the removal of impacted 3rd molars, some studies described a positive effect of laser, but others did not [26]. Therefore, until now, the parameters of optimal LLLT for biostimulation have not been known [29].

Clokie et al. [30], Fernando et al. [31], and Taube et al. [32] examined the effect of LLLT application on pain and swelling after the removal of the bilateral lower 3rd molar in the same surgical procedures, although Roynesdal et al. [33] examined the effect of LLLT application on swelling, pain, and trismus after the removal of the bilateral lower 3rd molar in two separate surgical procedures. The researchers used different laser parameters in these studies; all suggested that LLLT had no beneficial effect on swelling and trismus after extraction of the wisdom 3^rd^ molar. Clokie et al. [30] reported that there was a statistically significant difference in the reduction of pain on the day of surgery and on the 1st postoperative day. In our study, we found that LLLT was effective in decreasing pain levels only on the 7th postoperative day. However, Carillo et al. [34] described that although there was a statistically significant difference in the decrease in the ratio of trismus in the laser group up to 7 days after surgery, there were no differences in the percentage of swelling and pain between the laser and placebo groups.

Aras et al. [35] investigated the impact of intraoral and extraoral applications of LLLT on swelling and trismus after the removal of mandibular 3rd molars. 48 patients were divided into 3 equal groups (16 each); as follows: extraoral LLLT, intraoral LLLT, and placebo. They used the GaAlAs diode laser device with a continuous wavelength of 808 nm in their study and applied laser energy at 100 mW (0.1 W) for a total of 120 s (12 J). They found that use of LLLT extraorally had a significantly positive effect on trismus and swelling. Kazancioglu et al. [36] examined and compared the effect of LLLT and ozone therapy after wisdom 3rd-molar surgery by applying 12 J (4 J/cm^2^) of energy with a GaAlAs diode laser at 808 nm extraorally immediately after the surgical procedure and on the postoperative 1st, 3rd, and 7th day in the laser group. They reported that the pain level was lower in the ozonated and LLLT applied groups than in the control group; however, trismus and swelling in the LLLT group were significantly lower than in the ozonated and control groups. Acar et al. [37] evaluated the efficacy of LLLT and low-intensity pulsed ultrasound (LIPUS), alone and in combination, in triggering new bone formation. They demonstrated the efficacy of LLLT or LIPUS in triggering bone regeneration. Lim et al. [38] investigated in vitro effects of low-intensity pulsed ultrasound stimulation on the osteogenic differentiation of human alveolar bone-derived mesenchymal stem cells (hABMSCs) for tooth tissue engineering. This study reported that LIPUS could enhance the cell viability and osteogenic differentiation of hABMSCs, and could be part of effective treatment methods for clinical applications. Ferrante et al. [26] studied 2 groups that treated removal of a lower 3rd molar, by applying 54 J of energy with a laser diode at 980 nm intraorally and extraorally immediately after surgery and at 24 h. They recorded the number of days and levels of postoperative pain. The statistical analysis showed significant differences between the laser group and the control group in terms of swelling and trismus, but there was no significant difference in terms of pain levels. These authors observed that LLLT was more effective when it was applied extraorally instead of intraorally. In contrast to these results, in our study, although LLLT was applied extraorally, there was no statistically significant difference in terms of trismus and swelling levels, but there was a significant decrease in the pain level on the 7th day.

The mechanism of the analgesic effect provided by LLLT is not yet certain. There is evidence that LLLT has significant neuropharmacological effects on the synthesis, release, and metabolism of such neurochemicals as serotonin and acetylcholine at the central level and histamine and prostaglandin at the peripheral level. This analgesic effect can be explained with the effect of LLLT on the synthesis of endorphin and the decrease in the activity or bradykining of C fibers [39].

Trismus, degree of inflammation, or pain intensity may differ among patients. Thus, the separate surgical procedure design of this study helped avoid bias in the data collection [29], different from when the experimental individuals and the controls are different [26, 34, 35]. This study was managed with similarly impacted lower 3rd molar tooth with an equal grade of difficulty; thus, each person was his or her own control.

Dimensional measurements were made in the assessment of the swelling that occurred after the surgical procedure in previous studies. The 3dMD Face method was used in our study in order to assess the volumetric increase in swelling. With this method, the 3dMD image of the patient was taken before the surgery (0) and on the 2nd and 7th days after the surgery, and the volume of the area between the 2 images was calculated using the 3DMD program by aligning the images from the 2nd day and day 0, and the 7th day and day 0. We believe that the assessment made using this method yields a more accurate result.

## Conclusions

The procedures and outcomes of previous studies are too varied to describe the perfect parameters for use of LLLT or to assess its clinical efficiency. In this study, LLLT was applied extraorally and furthermore we used a different method to evaluate objectively volume changes. Although the results indicate that the proposed method reduces pain, swelling, and trismus, significant differences were observed only in the 7^th^ day pain level in the laser group compared with the control group.

## Abbreviations

3D, three-dimensional; GaAlAs, gallium–aluminum–arsenide; LLLT, low-level laser therapy; MMO, maximum mouth opening; VAS, visual analogue scale
